# Leukogram Profile and Clinical Status in* vivax* and* falciparum* Malaria Patients from Colombia

**DOI:** 10.1155/2015/796182

**Published:** 2015-11-18

**Authors:** Alberto Tobón-Castaño, Esteban Mesa-Echeverry, Andrés Felipe Miranda-Arboleda

**Affiliations:** Grupo Malaria, Facultad de Medicina, Universidad de Antioquia, Calle 70, No. 52-21, Medellín, Colombia

## Abstract

*Introduction*. Hematological alterations are frequent in malaria patients; the relationship between alterations in white blood cell counts and clinical status in malaria is not well understood. In Colombia, with low endemicity and unstable transmission for malaria, with malaria* vivax* predominance, the hematologic profile in malaria patients is not well characterized. The aim of this study was to characterize the leukogram in malaria patients and to analyze its alterations in relation to the clinical status.* Methods*. 888 leukogram profiles of malaria patients from different Colombian regions were studied: 556 with* P. falciparum* infection (62.6%), 313 with* P. vivax* infection (35.2%), and 19 with mixed infection by these species (2.1%).* Results*. Leukocyte counts at diagnosis were within normal range in 79% of patients and 18% had leucopenia; the most frequent alteration was lymphopenia (54%) followed by monocytosis (11%); the differential granulocyte count in 298 patients revealed eosinophilia (15%) and high basophil counts (8%). Leukocytosis, eosinopenia, and neutrophilia were associated with clinical complications. The utility of changes in leukocyte counts as markers of severity should be explored in depth. A better understanding of these hematological parameters will allow their use in prompt diagnosis of malaria complications and monitoring treatment response.

## 1. Introduction

Malaria, a disease caused by protozoan parasites of the genus* Plasmodium*, is still considered a serious global public health problem [1]. There were approximately 198 million cases and 584,000 deaths in 2013, according to the World Health Organization (WHO) [2]; however it has been noted in recent years that there is significant underreporting of cases and deaths worldwide [3]. Fatal cases are mainly attributed to* Plasmodium falciparum*; however other plasmodia, like* P. vivax,* can cause serious illness and even death [4, 5].

Malaria can affect any organ or system, the hematological system being the most affected one. The majority of described alterations occur on the red cell line and platelets, causing anemia and thrombocytopenia [6–8]. Alterations in the white blood cell (WBC) counts are less reported and have been associated with factors such as severity,* Plasmodium* species, concurrent infections, and treatment response [9–12]. Descriptions of leukocytosis, leukopenia, neutrophilia, and neutropenia, presence of immature neutrophils, and changes in lymphocytes, monocytes, and eosinophils in these patients have been observed [13, 14], but studies have not shown a specific leukocyte profile alteration in malaria.

It has been found that WBC counts are generally lower in malaria patients compared to healthy patients and that there is a trend towards lower WBC counts in patients infected with either* P. falciparum* or* P. vivax* [15]. Additionally it has been observed that these changes are dynamic during illness, with lower lymphocyte and eosinophil counts at baseline and higher counts as the symptoms disappear [15, 16]. There have been descriptions of mortality-associated leukocytosis, presence of coinfections, and gram negative bacteremia in complicated cases of* P. falciparum* malaria [17].

Several theories attempt to explain these described hematologic disorders, including (i) bone marrow (BM) suppression secondary to immune response imbalances, (ii) lower average cell life, and (iii) leukocyte redistribution to lymphoid organs and tissues with increased inflammatory response or sequestration in microvasculature [11, 14, 18–20]. However, these findings remain poorly understood and their utility in clinical practice has not yet been established, unlike for other infectious diseases prevalent in tropical areas, such as Dengue [21].

The Complete Blood Count (CBC) assesses the cellular elements of the blood, that is, red cells, white cells, and platelets, both qualitatively and quantitatively [22] and is an essential tool in assessing hematological changes. The leukogram is part of the CBC that analyzes white blood cells; it comprises the total WBC and subpopulation counts including neutrophils, eosinophils, basophils, lymphocytes, and monocytes. Their reference values vary according to age, race, physiological condition (pregnancy), use of some drugs, and time of day [23]. The aim of this study is to characterize changes in the WBC count of patients with malaria from endemic regions in Colombia and to analyze variations according to clinical status, species of* Plasmodium*, and some sociodemographic characteristics of the patients. Our purpose is to encourage the use of easily accessible diagnostic tools, such as the CBC, allowing early identification of patients at risk for clinical complications and to implement appropriate therapeutic measures, improving patient outcome.

## 2. Methods and Materials

A descriptive retrospective analysis was made with clinical, laboratory, and demographic data from outpatients with* P. falciparum* or* P. vivax* malaria, from endemic regions in Colombia. The reference population consisted of patients of all ages with symptoms suggestive of malaria, with parasitological diagnosis confirmed with thick blood smear examination in first or second level of care hospitals and health care facilities in the municipalities of Turbo and Necoclí (Urabá, Antioquia), El Bagre (Bajo Cauca, Antioquia), Tumaco, Guapi, and Timbiquí (Pacific Coast), and who were enrolled in clinical and epidemiological studies conducted by the Malaria Group between 1997 and 2007 [24–26]. We also included Medellín third-level hospitals' inpatients' data from 2005 to 2010, referred from different regions. Convenience sampling was performed for this analysis; only patients with a CBC performed at hospital admission were included, obtaining a sample of 888 patients.

The thick smear examination was done according to WHO recommendations [27]. After a medical assessment, a sample of blood was taken in EDTA for cellular and biochemical studies, including a CBC within 2 hours, obtained in 3rd- and 4th-generation automated analyzers (Abbott Diagnostics Cell-Dyn3200; Beckman Coulter, Coulter HmX; and Nihon Kohden Celltac MEK, Sysmex KX-21N). Colombian population reference values for the WBC counts were applied (Table 1) [28].

The WBC count, hemoglobin values, and platelets counts were qualitatively classified as low or high if they were above or below the normal range. Complications were classified according to WHO major criteria for severity [29] and to proposed minor criteria for Colombia [26] as follows:

(1) liver failure: severe liver failure (total bilirubin >3 mg/dL; AST >120 IU/L) or mild liver failure (total bilirubin > 1.5–3 mg/dL; AST > 80–120 IU/L); (2) thrombocytopenia: profound (<25,000 platelets/*μ*L) or severe (25–50,000 platelets/*μ*L); (3) renal impairment: severe (BUN > 60 mg/dL or creatinine > 3 mg/dL) or mild (BUN 41–60 mg/dL or creatinine 1.5–3 mg/dL); (4) anemia: severe (hemoglobin < 5 gr/dL) or moderate (hemoglobin 5–6.9 gr/dL); (5) neurologic complication: cerebral malaria (seizures/coma) or extreme weakness; (6) pulmonary injury: acute respiratory distress syndrome (ARDS)/pulmonary edema or pleural effusion; (7) acid-base disturbance: severe acidosis (pH < 7.35 and HCO_3_ < 15 mEq/L) or acidosis (pH < 7.35 and HCO_3_ 15–18 mEq/L); and (8) hypoglycemia: severe (<40 mg/dL) or moderate (40–49 mg/dL).

Patients were divided into five age groups for analysis. Parasitemia was classified by ranges of parasites/*μ*L in up to 1,000; 1,001–5,000; 5,001–10,000; 10,001–50,000; 50,001–100,000; and greater than 100,000.

### 2.1. Statistical Analysis

The Kolmogorov-Smirnov test was used to assess normality for quantitative variables; the median values were compared with Mann-Whitney *U* test when variables were not normal. Associations between qualitative variables were analyzed with the *χ* ^2^ test. Spearman's rank correlation coefficient was applied to analyze some relationships between numerical data. The adopted level of statistical significance was 5% (*P* value <0.05). The analysis was done with IBM SPSS statistics program, 22nd version (licensed to Universidad de Antioquia).

### 2.2. Ethical Aspects

The patients studied prospectively were included after giving informed consent, endorsed by the Ethics Committee of the Medical Research Institute from the Medical School of the Universidad de Antioquia (Minute 31, July 2002; Minute 5, June 2005). The review of retrospectively studied patients' medical records was approved by the Hospital Ethics Committees from Hospital Pablo Tobón Uribe and Hospital San Vicente Fundación (Letter 5282, October 2009).

## 3. Results

**Figure 1:**
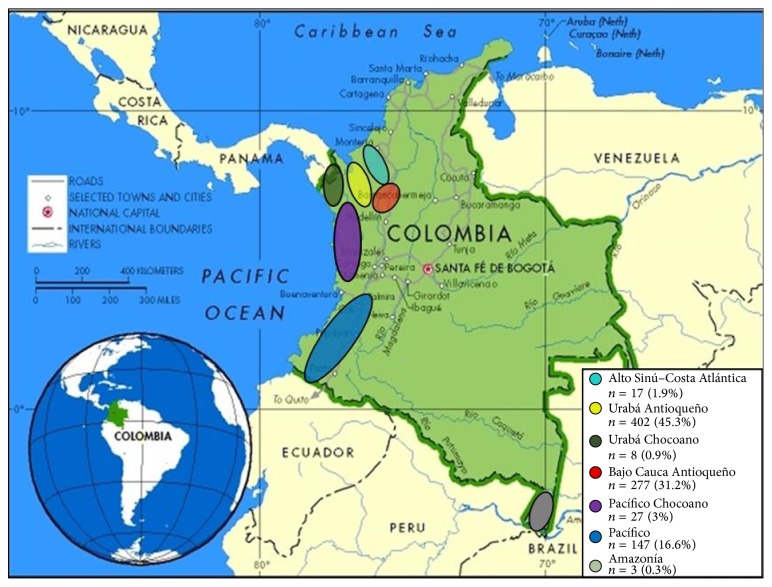
Geographical location of patients' residence by Colombian malarial endemic regions. Obtained and modified from the Cartographic Design Unit of the World Bank, 2000.

Blood counts of 888 patients were analyzed. The quantitative variables showed normal distribution, except hemoglobin (KS, *P* = 0.082). The sample consisted mostly of men (62.8%) over 15 years of age, with a mean age of 26 years (median 22.8). Age ranged from 1 month to 82 years. The geographic origin of the patients is shown in Figure 1. Some demographic characteristics are described in Table 2.

The time of disease progression to first medical consultation was known for 618 patients: 30% (*n* = 185) consulted within the first three days of onset of symptoms, 44.9% (*n* = 277) between days 4 and 7, and 21.3% (*n* = 132) from day 8 to 15; 3.8% of the study population consulted 2 weeks after the onset of symptoms. Previous use of antimicrobials was established (not antimalarial) in 39 (4.4%) patients (particularly penicillins in 23 and cephalosporins in 7) and antimalarials in 79 (8.9%) (particularly chloroquine and primaquine in 68 and quinine in 8). Complicated malaria was diagnosed in 257 patients (29%): 110 (12%) with major criteria and 147 (16.6%) with minor criteria for severity; no deaths were reported.

### 3.1. Parasite Counts Description

*P. falciparum* infection was diagnosed in 556 patients (62.6%);* P. vivax* was in 313 (35.2%) and mixed infection by these species in 19 (2.1%). The median parasite count in 809 patients was 4,980/*μ*L; 71.8% (*n* = 581) had counts lower than 10,000 parasites/*μ*L and 1% (*n* = 8) had parasitemia above 100,000/*μ*L. In parasitemia ranges, 19.9% had less than 1,000 parasites/*μ*L, 51.8% between 1,000 and 10,000, 24.5% between 10,001 and 50,000, and 2.7% between 50,000 and 100,000.

### 3.2. Leukogram Findings

**Figure 2:**
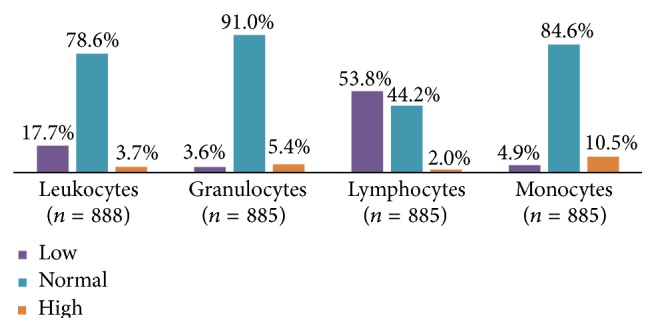
Leukocyte counts distributions.

**Figure 3:**
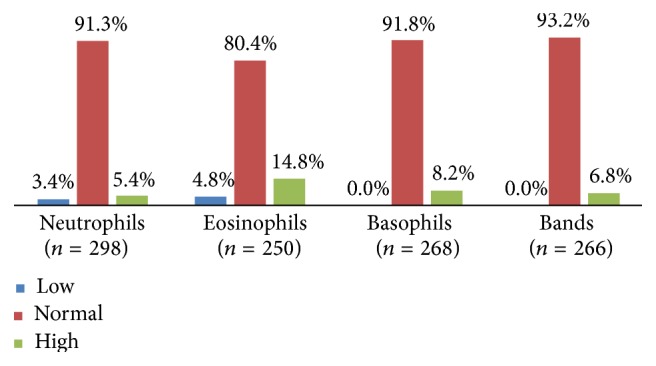
Granulocyte subpopulations counts.

Leukocyte subpopulations were not normally distributed (KS, *P* < 0.001); leukocyte counts at diagnosis were within the normal range in 79% (*n* = 698) of patients and 18% (*n* = 157) had leucopenia; the median value for these cells was 6,100/*μ*L. Figures 2 and 3 show subpopulation frequency distribution. Patients with previous use of antimicrobials had a higher frequency of leukopenia than those not taking any (35.9% versus 16.8%) (OR = 2.9; 1.4–5.7; *P* = 0.002); the former had lower leukocyte counts than the latter (Mann-Whitney *U* test; *P* = 0.016).

*Mononuclear Cells*. Lymphopenia prevailed in 54% (*n* = 476); the median value for these cells was 1,575/*μ*L. Monocyte count was normal in 85% of cases (*n* = 749); the median count was 371 cells/*μ*L.

*Granulocytes (PMN)*. Some of the analyzed blood counts did not have differential granulocyte subpopulations counts and only had a total granulocyte count. Normal values were found in 91% of patients (*n* = 805). One-third of differential counts came from severe patients and the rest from nonsevere ones (66%)

Analyzed neutrophils in 446 patients showed normal values in 91% of cases (*n* = 407); the median value was 3,836 cells/*μ*L. Only 250 patients had eosinophil counts of which 80% (*n* = 201) were normal and 15% (*n* = 37) had eosinophilia; the median value for these cells was 176/*μ*L. Eosinopenia was present in 5% (*n* = 12) of patients. Only 268 patients had basophil counts, of which 92% (*n* = 246) were within normal values. No patient in this group had counts below normal levels.

#### 3.2.1. Age of Patients

The median neutrophil value was lower in children; there was greater variability in their counts with the highest individual neutrophil counts. Means and medians are presented in Table 3. No significant differences between groups were observed when values were assigned into the categories of normal, low, or high (*P* > 0.05). Children were more likely to have monocytosis (RR = 2.2, 1.4–3.5; *P* < 0.001), neutrophilia (RR = 2.3, 1.0–5.2; *P* = 0.046), and eosinophilia (RR = 2.2, 1.1–4.5; *P* = 0.029) than adults (Table 4).

#### 3.2.2. Parasitemia and* Plasmodium* Species

There was a negative relationship between the number of parasites and the eosinophil count (Spearman coefficient −0.2; *P* = 0.010); no linear correlation was found with other cell populations. There were no differences in leukocyte subpopulations medians in relation to plasmodial species (*P* > 0.05) (Table 3).

#### 3.2.3. Severe Malaria

Neutrophilia and eosinopenia were the most common alterations in severe malaria cases. The comparison between severe malaria (overall complications, diagnosed with major or minor criteria) and nonsevere malaria shows that leukopenia and leukocytosis, lymphopenia, eosinopenia, monocytosis, and neutrophilia were linked to severity (Table 5). These associations remained when only major criteria were applied, except for leukocytosis. When only minor criteria were applied, leukopenia (OR = 1.7; 1.1–2.6; *P* = 0.022), leukocytosis (OR = 2.5; 1.01–2.6; *P* = 0.039), lymphopenia (OR = 2.2; 1.5–3.2; *P* < 0.001), and eosinopenia (OR = 10.8; 2.1–55.6; *P* = 0.002) were associated with severity. Leucocyte counts are compared in relation to clinical condition in Table 6.

#### 3.2.4. Glycemia and Leukocytes

Glycemia values were obtained from 357 subjects, averaging 107.6 mg/dL (37–264) with a median of 102 mg/d. When glycemia was compared with leukocyte counts, patients with leucopenia had significantly higher blood glucose with a median of 108 mg/dL and those with normal WBC count had a median of 101 mg/dL (*P* = 0.033, Mann-Whitney); leukopenia patients had an increased risk of elevated blood sugar levels (>140 mg/dL) (OR = 2.6; 1.4–5; *P* = 0.004) compared with patients with a normal WBC count.

Lymphopenia patients had a median blood glucose of 109 mg/dL versus 95 mg/dL in patients with normal lymphocyte counts (*P* < 0.001, Mann-Whitney); lymphopenia was associated with an increased risk of high blood glucose levels (>140 mg/dL) (OR = 2.8; 1.3–5.7; *P* = 0.007).

Low granulocyte counts were related to lower blood glucose values, with a median of 81 mg/dL versus 103 mg/dL in patients with normal granulocyte counts (*P* = 0.002, Mann-Whitney), but the values remained within normal ranges. There was no higher risk of high blood glucose (*P* < 0.05) in patients with low granulocyte counts.

#### 3.2.5. Leukocytes and Other CBC Variables

A direct correlation between the WBC and platelet counts (Spearman coefficient 0.27, *P* < 0.001) was found. This correlation is maintained with lymphocytes (0.27; *P* < 0.001), monocytes (0.14; *P* < 0.001), granulocytes (0.18; *P* < 0.001), neutrophils (0.12; *P* = 0.016), and eosinophils (0.25; *P* < 0.001). The median platelet count differed significantly between patients with leukopenia (100,000/*μ*L; *n* = 154) and those without leukopenia (131,000/*μ*L; *n* = 704) (*P* < 0.001, Mann-Whitney). Thrombocytopenia defined as a platelet count <150,000/*μ*L was a risk factor for developing leucopenia, both with (OR = 5.8; 2.7–12.4) and without anemia (OR = 3.7; 0.9–7.4). Patients with severe thrombocytopenia had significantly lower leukocyte counts if infected with* P. vivax* (median 4,850 cells/*μ*L) compared to* P. falciparum* infections (median 6,100 cells/*μ*L) (*P* = 0.011, Mann-Whitney), a difference that was not observed by species of* Plasmodium* among patients without thrombocytopenia.

No correlation between leukocyte and erythrocyte counts was found (Spearman = 0.032; *P* = 0.364); the median erythrocyte count did not differ (*P* = 0.3133; Mann-Whitney) between patients with leucopenia (4,365,000/mm^3^; *n* = 142) and those without leukopenia (4,400,000/mm^3^; *n* = 620); hemoglobin values did not differ between patients with leukopenia (11.6 mg/dL; *n* = 157) and those without leukopenia (12.1 mg/dL; *n* = 698) (*P* = 0.164, Mann-Whitney). A negative correlation between lymphocyte and erythrocyte counts (Spearman = −0.18; *P* < 0.001) and a positive one between lymphocyte and neutrophils counts (Spearman = 0.18; *P* < 0.001) were found.

A negative relationship between the number of parasites and the eosinophil count was found (Spearman coefficient −0.18; *P* < 0.001); no linear correlation was found with other cell populations.

## 4. Discussion

Of the studied patients, 70% consulted after three days of febrile syndrome onset and 66% of the blood counts were performed between days 4 and 15 of the disease; thus WBC counts may reflect the response to repeated erythrocytic schizogony. One of the difficulties in establishing a pattern of leukocyte alteration during malaria episodes is the presence of different infection stages when the WBC count is analyzed. This variation in hematological parameters is expected as days pass from the onset of infection and may also be the result of drug administration as observed with the use of antimicrobials in these patients.

The prevalent* Plasmodium* species in the cases studied was* P. falciparum*, in 63% of patients; this differs from the epidemiology of malaria in Colombia where the predominant species is* P. vivax*, a situation possibly due the fact that more* falciparum* cases require hospitalization. Parasitemia ranges were below 10,000 parasites per microliter in 72% of patients (approximately less than 0.25% of parasitized erythrocytes). This variable was not associated with leukocyte alteration, except for the negative correlation between parasitemia and eosinophil counts (Spearman, *P* < 0.001).

The leukogram profile was similar in* P. vivax* and* P. falciparum malaria*. Although a strong inflammatory response in* P. vivax* infections has been recognized [30], our study did not find a differential leukocyte response by* Plasmodium* species, except for patients with severe thrombocytopenia and* P. vivax* infection, whose leukocyte counts were significantly lower, in accordance with previous findings in the acute phase of infection [31]. Leukopenia is a common finding in* falciparum* and* vivax* malaria [15, 16, 32]. Although some studies indicate that there is a greater trend towards leukopenia in infections with* P. falciparum*, this also happens with* P. vivax* [15, 33, 34]. Therefore the presence of leukopenia or its severity does not seem to be useful for diagnosis of species.

Although* Plasmodium* sp. is a hemoparasite, it has been observed that malaria does not significantly affect WBC counts, with values that are usually normal for total leukocyte population for both granulocyte and mononuclear cell subpopulations. Publications on leukocyte alterations in patients with malaria mostly reported WBC counts within normal ranges [9, 32–36]. These findings are consistent with our results where 85% of patients had normal leukocyte counts. The leukocyte, lymphocyte, monocyte, and neutrophil counts differed significantly between children and adults, but the values were within normal limits; therefore these differences appear to be due to physiological variability between groups.

Some changes have been reported in the distribution of WBC lines during the acute phase of malaria infection as well as the return to normal values during the convalescent phase [31], and there is a clear normalizing trend of WBC counts after disease resolution [15], meaning that WBC counts could be used as an indicator for the progress of disease, providing guidance for its management.

### 4.1. Leukopenia

Leukopenia is an alteration present in both* falciparum* and* vivax* malaria [15, 16]. Our study found that leukopenia was more frequent (18%) than leukocytosis (4%). This is the most reported alteration in malaria with frequencies up to 22% [13, 34, 36, 37]. Leukopenia (WBC < 6,100 cells/*μ*L) was present in 10.2% [17] of Kenyan children over 3 months of age, hospitalized with* P. falciparum* malaria. Using a cutoff value of <4,000 cells/*μ*L to define leukopenia, this alteration was observed in 22.1% of infections by* P. vivax* and 18.4% of mixed infections [37]. In experimental infections with* P. falciparum*, counts under 3,000 cells/*μ*L were identified in 12% (*n* = 83) and counts under 1,000 cells/*μ*L were in 9% (*n* = 79) [38]. In the UK leukopenia was described in 7% of patients with imported* P. falciparum* malaria [39].

Leukopenia in malaria can be caused by the interaction of various events. It has been proposed that the sequestration of leukocytes causes the decline in WBC counts more than a decreased production or accelerated destruction [11]. Glycosylphosphatidylinositol (GPI), an immunogenic antigen common to all species of* Plasmodium*, stimulates the production of proinflammatory cytokines in monocytes and macrophages [15, 40]; this would increase phagocytosis generating cell debris and increasing RBC and WBC phagocytosis, thus maintaining increased proinflammatory cytokine levels, particularly TNF-*α*, causing altered hemopoiesis [15, 41, 42].

### 4.2. Leukocytosis

Leukocytosis frequency in malaria varies from one study to another [11–13, 39] and is reported predominantly in* falciparum* malaria. This alteration was identified in 4% of our patients, without statistically significant differences by* Plasmodium* species (*P* > 0.05); the presence of coinfections that may explain leukocytosis was not studied.

Leukocytosis occurs mainly by increased PMN numbers, but lymphocytosis, monocytosis, and eosinophilia can also occur [15, 17, 34]. A study of the cellular composition of BM [9] concluded that during* Plasmodium* infection there was a decrease in some core precursors and in PMN reserves, with increased circulation of immature PMN (bands) and abnormalities in their response to stimuli with drugs and endotoxin; the initial stimulus of infection on the BM reserves and increased circulating half-life of PMN lead to a transient leukocytosis. In* falciparum* malaria, leukocytosis has been explained by the stimulus of infection on the BM to release leukocytes during the paroxysms [16]; increased proinflammatory cytokines could favor the exit of leukocytes from BM [14] or by the presence of bacterial or viral coinfections [12], considering that these possible diagnoses were not excluded in some of the studies [17].

Comparative studies between severe and nonsevere malaria point out leukocytosis as a marker of severity and a predictor of death. A WBC count greater than 16,500 cells/*μ*L was associated with a 7.14 times greater risk of death; mortality was related to the occurrence of bacteremia, polypnea, and coma [17]. When comparing severe and nonsevere patients, leukocytosis was found in 19% of severe and 12.7% of uncomplicated malaria patients; leukocytosis patients were younger and had other manifestations of severe malaria such as hypoglycemia and severe anemia and a 3.5 times greater risk of dying [12]. In our study 33 patients had leukocytosis and 42% of them had some complications (OR = 2.01; 1.1–4.2; *P* = 0.040); specifically renal dysfunction was related to this alteration.

### 4.3. Lymphocytes

Lymphopenia was the most common abnormality in our patients, present in 54% of cases, while only 2% had lymphocytosis; it was associated with thrombocytopenia as were lymphopenia and neutropenia (*P* < 0.001). In the acute-stage malaria infection lymphopenia (defined as an absolute lymphocyte count ≤1500 cells/*μ*L) was common in* vivax* and* falciparum* cases [15]. This finding was similarly reported in 63% of patients with imported* falciparum* malaria; and along with thrombocytopenia it represented the most common alteration [39].

The etiology of lymphopenia is open to discussion; it has been proposed that during the onset of infection lymphocyte counts drop due to lymphocyte stimulation through inflammation and their following distribution to more active tissues [43] followed by a reorganization of cellular distribution after disease resolution with a return to normal values [44].

A study in animal models found, however, significant elevations of serum apoptotic factors such as Fas and FasL in macaques, which were more susceptible to malaria complications and developed a more pronounced lymphopenia at the expense of T cells [45]; these findings were subsequently confirmed in humans with* P. falciparum* malaria: samples from these patients showed higher values of the aforementioned apoptotic factors at diagnosis and a progressive decrease as the treatment advanced, coinciding with an increase in lymphocyte counts [46]. Despite these results, a clear correlation between elevated Fas-FasL and decreased lymphocytes counts has not been established [47] and both are believed to be the result of malaria immune hyperstimulation causing lymphocyte downregulation to maintain cellular homeostasis or a strategy of the* Plasmodium* genus parasites to decrease immune response [48]. Sequestration of lymphocytes in organs such as the spleen, presence of atypical or plasmacytoid lymphocytes, or decreased survival of these cells have been described [14, 15, 19].

Lymphopenia is a complex pathogenic phenomenon that may have important clinical effects in patients because of the transient immunodeficiency state that can occur and the increased risk of complications [44]; lymphopenia in our patients was associated, in general, with severity and specifically with moderate or severe hepatic dysfunction.

### 4.4. Monocytes and Granulocytes

Reported changes in other WBC like monocytes and eosinophils are less frequent; eosinopenia has been reported at malaria diagnosis [14–16], but, overall, there has been full recovery during the course of the disease, returning to normal values or even tending to mild eosinophilia at the end of treatment [16, 48]. In our patients 85% of monocyte and 91% of granulocyte counts were normal. Monocytosis presented in 11% of cases and it was associated with severe anemia, in line with the above findings for lymphocytosis. Although it has been described that PMNs are largely responsible for the elevation of leukocyte counts, a strong association of monocytosis and lymphocytosis with mortality has been highlighted [17].

Eosinophilia was the most frequent alteration in granulocytes (15% of 250 patients) while eosinopenia was identified in 5% of cases, which was associated with severe malaria and specifically with neurological, pulmonary, and renal complications and with severe thrombocytopenia. Low eosinophil levels have been reported in the early stage of acute* falciparum* and* vivax* infections (normal values ≤350 cells/*μ*L), with increasing levels over the following few weeks [15]. Increased eosinophil counts have been associated with better recovery from malaria anemia secondary to allergies caused by drugs [16] and have also been proposed as an indicator of adequate response to treatment in malaria patients [14, 15].

Neutrophil counts were normal in 91% of cases, with neutrophilia prevailing over neutropenia (5% versus 3%); it was associated with severe malaria and specifically with severe hypoglycemia and moderate or severe liver and kidney dysfunction. Altered basophil counts were not associated with severity. Although differential granulocyte counts were not available for all patients, 33% came from severe cases, which ensures inclusion of complicated and noncomplicated patients in the analysis.

### 4.5. Leukocytes, Erythrocytes, and Platelets

Leukocyte changes were not related to the decrease in erythrocyte counts and hemoglobin, except for the relation of monocytosis and lymphocytosis with severe anemia in 26 patients. It is possible that a larger number of cases of severe anemia allow identifying other abnormalities in the WBC count. Platelet count, by contrast, positively correlated with changes in all the white lines (Spearman correlation, *P* < 0.05) and total leukocyte lymphocyte, monocyte, and eosinophil counts were lower in patients with severe thrombocytopenia though only eosinophils showed statistical significance. These findings suggest an active role of leukocytes in platelet sequestration sites, via cellular aggregation, a phenomenon described for platelets [49] and leukocytes [50].

### 4.6. Leukocytes and Glycemia

A trend of increasing blood glucose was observed in patients with leucopenia and the frequency of hyperglycemia was higher, with statistical significance; specifically lymphopenia was associated with higher blood sugar levels. On the contrary, neutrophilia was associated with severe hypoglycemia, a complication occurring in only 4 patients.

## 5. Conclusions

Malaria hematological changes are diverse and can vary according to study,* Plasmodium* species, age, gender, and other factors. Although some studies have recognized the relationship between leukocytosis and clinical complications in malaria, the general picture involving the role of leukocytes that may play a key role is not entirely clear. The few studies that analyze the leukogram profile in malaria do not delve into the relationships of the leukocyte counts and the clinical condition of patients. This is one of the largest series of leukograms published in an attempt to explain the usefulness of this information as an indicator of severity.

The leukogram profile was similar in* P. vivax* and* P. falciparum malaria*. Leukocyte counts were within normal range in 79% of patients and 18% had leucopenia; the most frequent alteration was lymphopenia (54%) followed by monocytosis (11%). In this study, alterations in leukocyte counts were related to clinical complications according to major criteria of severity; specifically lymphopenia, eosinopenia, monocytosis, and neutrophilia were indicators of serious injury. Leukopenia, lymphopenia, and eosinopenia were related to complications in patients with minor criteria, probably indicating that leukocyte changes begin early and may suggest the onset of serious injury.

Despite the limitations related to retrospective analysis, this study contributes to a better compression about the leukocyte response to plasmodial infection and its relation with the clinical status. The blood samples were processed in 3rd- and 4th-generation automated analyzers; this reflects the technological level and the devices that are available in endemic regions, used to make treatment decisions. Taking into account the limitations of the information provided by these devices, our findings' information could be used to recognize clinical deterioration and to provide adequate care and support.

This study determined changes in the WBC count of malaria patients and analyzed variations according to clinical status. Some alterations in the WBC profile were related to clinical severity and could allow early identification of patients at risk for medical complications. Their utility as markers of severity should be explored in depth, in addition to their application, to identify disease resolution. A better understanding of these hematological parameters will allow their use in early diagnosis of malaria complications and monitoring treatment response.

## Figures and Tables

**Table 1:** Leukogram reference values by age^*∗*^.

Age	Leukocytes/*µ*L	Neutrophils/*µ*L	Lymphocytes/*µ*L	Monocytes/*µ*L	Eosinophils/*µ*L	Basophils/*µ*L
1–15 days	9,000–30,000	1,000–12,000	3,000–9,000	30–750	40–500	0–50
16–31 days	5,000–21,000	1,000–12,000	3,000–9,000	30–750	40–500	0–50
1–12 months	6,000–17,500	1,000–12,000	3,000–9,000	30–750	40–500	0–50
1–5 years	5,500–15,500	1,700–7,500	3,000–9,000	30–750	40–500	0–50
6–14 years	4,500–14,500	1,500–6,500	2,000–7,200	30–750	40–500	0–50
15–99 years	4,500–11,000	1,500–8,000	1,500–4,000	30–900	40–500	0–50

^*∗*^Leukogram counts based on gender not shown.

Adapted from [28].

**Table 2:** Demographic characteristics of 888 malaria patients from Colombia.

Characteristics	*n*	%
Gender		
Male	558	63
Female	330	37
Age		
Less than 1 year	8	0.9
1.1 to 5 years	50	5.6
5.1 to 15 years	193	21.7
15.1 to 65 years	624	70.3
Over 65 years	13	1.5

**Table 3:** Leukocyte subpopulations (cells/*µ*L) by age ranges and malaria etiology.

		Less than 1 year (*n* = 8)	1.1 to 5 years (*n* = 50)	5.1 to 15 years (*n* = 193)	15.1 to 65 years (*n* = 624)	Over 65 years (*n* = 13)	*P. falciparum* ^*∗∗*^ (*n* = 556)	*P. vivax* (313)
Leukocytes (*n* = 888)	Mean	13,238	8,360	6,723	6,438	6,671	6,597	6,700
	CI^*∗*^	6,592–19,883	7,480–9,240	6,289–7,158	6,250–6,627	5,676–7,667	6,385–6,810	6,414–7,086
	Median	10,050	8,200	6,200	6,000	6,000	6,000	6,200

Lymphocytes (*n* = 885)	Mean	5,914	3,803	2,037	1,639	1,427	1,897	1,850
	CI	3,450–8,377	3,251–4355	1,840–2,233	1,564–1,715	1,034–1,820	1,786–2,007	1,707–1,993
	Median	5,472	3,473	1,776	1,451	1,248	1,585	1,539

Monocytes (*n* = 885)	Mean	776	663	462	425	436	434	454
	CI	193–1,360	479–846	408–516	398–452	296	402–466	415–494
	Median	652	434	383	348	576	360	372

Granulocytes (*n* = 885)	Mean	6,516	3,887	4,188	4,370	4,829	4,266	4,420
	CI	1,422–11,610	3242–4533	3880–4496	4207–4532	3,772–5,885	4,098–4,435	4,164–4,675
	Median	4,502	3,590	3,768	4,128	4,210	3,950	4,015

Neutrophils (*n* = 446)	Mean	4,791	3,753	3,886	4,264	4,585	4,087	4,225
	CI	2,375–7,208	2,736–4,770	3,522–4,250	4,028–4,501	3,277–5,893	3,838–4,336	3,948–4,501
	Median	4,947	3,432	3,565	3,950	3,869	3,780	3,956

Eosinophils (*n* = 250)	Mean	662	413	405	292	68	279	469
	CI	1,595–2,919	54–773	203–606	226–358	33–101	228–330	197–741
	Median	248	224	200	179	56	168	254

Basophils (*n* = 268)	Mean	4	54	8	15	88	12	35
	CI	9–17	8–117	3–13	2–28	138–315	5–18	−6–77
	Median	—	—	—	—	—	—	—

^*∗*^CI: 95% confidence interval. ^*∗∗*^No differences found in WBC counts when comparing between species.

**Table 4:** White blood cells per *µ*L in children and adults.

Cell group	Children	Adults
Leukocytes (medians)	6.650 (*n* = 252) (IR^*∗*^ = 3,675)	6.000 (*n* = 636) (IR = 2,800)
Lymphocytes (medians)	2.030 (*n* = 251) (IR = 1,835)	1.442 (*n* = 634) (IR = 1,109)
Monocytes (medians)	400 (*n* = 251) (IR = 3,675)	348 (*n* = 435) (IR = 351)
Neutrophils (medians)	3.603 (*n* = 130) (IR = 2,154)	3,927 (*n* = 316) (IR = 2,371)
Eosinophils (medians)	204 (*n* = 79) (IR = 375)	168 (*n* = 171) (IR = 261)
Basophils (mean value)	17 (*n* = 87) (CI^*∗∗*^ = 5–30)	18 (*n* = 181) (CI = 4–31)
Bands (mean value)	34 (*n* = 87) (CI = (−3)–70)	23 (*n* = 179) (CI = 11–36)

^**∗**^Interquartile ranges.

^*∗∗*^95% confidence interval.

**Table 5:** Comparison of leukocyte alterations between complicated and noncomplicated patients.

		Normal *n* (%)	Low *n* (%)	High *n* (%)
Leukocytes	Complication (no)	516 (73.9)	96 (61.1)	19 (57.6)
	Complication (yes)	182 (26.1)	61 (38.9)	14 (42.4)
	OR (CI), *P* ^**∗**^		1.8 (1.3–2.6), **0.001**	2.1 (1.0–4.2), **0.038**

Lymphocytes	Complication (no)	309 (79.0)	309 (64.9)	11 (61.1)
	Complication (yes)	82 (21.0)	167 (35.1)	7 (38.9)
	OR (CI), *P*		2.0 (1.5–2.8), **0.000**	2.4 (0.9–6.4), 0.071

Monocytes	Complication (no)	543 (72.5)	32 (74.4)	54 (58.1)
	Complication (yes)	206 (27.5)	11 (25.6)	39 (41.9)
	OR (CI), *P*		0.9 (0.5–1.8), 0.783	1.9 (1.2–3.0), **0.003**

Neutrophils	Complication (no)	278 (68.3)	11 (73.3)	10 (41.7)
	Complication (yes)	129 (31.7)	4 (26.7)	14 (58.3)
	OR (CI), *P*		0.8 (0.3–2.5), 0.680	3.0 (1.3–6.9), **0.007**

Eosinophils	Complication (no)	133 (66.2)	2 (16.7)	29 (78.4)
	Complication (yes)	68 (33.8)	10 (83.3)	8 (21.6)
	OR (CI), *P*		9.8 (2.0–93.2), **0.001** ^*∗∗*^	0.5 (0.2–1.2), 0.143

^**∗**^Odds ratio, confidence interval, and *P* value; ^*∗∗*^Fisher's exact test.

**Table 6:** Leucocyte counts (cells/*µ*L) in relation to clinical condition^*∗*^.

Groups	Leukocytes			Lymphocytes			Monocytes			Neutrophils			Eosinophils		
	*N*	Median	*P*	*N*	Median	*P*	*N*	Median	*P*	*N*	Median	*P*	*N*	Median	*P*
Complicated malaria															
Yes	257	6,100	0.365	256	1,358	**0.000**	256	347	0.545	147	4,030	0.114	86	111	**0.000**
No	631	6,200		629	1,680		629	378		299	3,952		164	242	
Hepatic dysfunction															
Yes	178	6,200	0.308	178	1,295	**0.001**	178	373	0.506	105	4,040	**0.005**	55	124	0.067
No	278	5,800		277	1,526		277	392		173	3,456		69	204	
Renal dysfunction															
Yes	34	7,400	**0.014**	34	1,422	0.949	34	457	0.081	26	5,079	**0.003**	19	158	0.605
No	421	5,900		420	1,449		420	386		253	3,550		105	158	
Neurological complication															
Yes	17	7,700	0.443	17	1,716	0.319	17	418	0.578	12	3,430	0.658	10	68	**0.012**
No	871	6,100		868	1,569		868	370		434	3,836		240	186	
Pulmonary complication															
Yes	17	7,700	0.425	17	1,716	0.218	17	418	0.267	17	4,235	0.560	10	85	**0.028**
No	871	6,100		868	1,569		868	370		868	3,954		240	186	
Severe hypoglycemia (<49 gr/dL)															
Yes	4	8,300	0.318	4	807	0.246	4	191	0.209	2	8,009	**0.004**	1	N.D.	0.147
No	352	5,950		351	1,425		351	366		194	3,672		67	204	
Severe anemia															
Yes	26	8,000	0.073	26	2,346	**0.000**	26	453	**0.025**	14	3,543	0.239	10	163	0.941
No	862	6,100		859	1,540		859	368		432	3,857		240	176	
Severe Thrombocytopenia															
Yes	93	5,700	0.084	93	1,372	0.069	93	273	0.058	55	3,950	0.918	44	93	**0.003**
No	765	6,200		762	1,587		762	382		361	3,822		190	205	
Acidosis															
Yes	12	8,200	0.501	12	1,191	0.091	12	437	0.687	12	5,502	0.170	11	154	0.092
No	20	6,250		20	1,432		20	768		20	3,285		18	73	

^**∗**^Major or minor criteria for severe malaria.
